# *Rickettsia monacensis* and Human Disease, Spain

**DOI:** 10.3201/eid1309.060186

**Published:** 2007-09

**Authors:** Isabel Jado, José A. Oteo, Mikel Aldámiz, Horacio Gil, Raquel Escudero, Valvanera Ibarra, Joseba Portu, Aranzazu Portillo, María J. Lezaun, Cristina García-Amil, Isabel Rodríguez-Moreno, Pedro Anda

**Affiliations:** * Centro Nacional de Microbiología, Majadahonda, Madrid, Spain; † Complejo San Millán-San Pedro de La Rioja, Logroño, Spain; ‡ Hospital de Txagorritxu, Vitoria, Spain

**Keywords:** Rickettsia monacensis, new pathogen, IrITA2, IrITA3, IRS3, IRS4, Ixodes ricinus, Mediterranean spotted fever, spotted fever group, Spain, dispatch

## Abstract

We identified *Rickettsia monacensis* as a cause of acute tickborne rickettsiosis in 2 humans. Its pathogenic role was assessed by culture and detection of the organism in patients’ blood samples. This finding increases the number of recognized human rickettsial pathogens and expands the known geographic distribution of Mediterranean spotted fever–like cases.

Tickborne rickettsioses are produced by spotted fever group (SFG) rickettsiae and cause an expanding spectrum of clinical signs. *Rickettsia conorii* is the etiologic agent of Mediterranean spotted fever (MSF) and is transmitted by *Rhipicephalus sanguineus*. *Rickettsia helvetica*, a widespread species, is carried by *Ixodes ricinus* (*1*). Recently, other SFG rickettsiae have been found in *I. ricinus* from Spain (*2*), Slovakia (*3*), and northeastern Italy (*4*), as well as in *I. nipponensis* from Japan (*5*). Subsequently, a new rickettsia species, *R. monacensis*, was isolated from *I. ricinus* from Germany (*6*) and detected in Hungary (*7*). The pathogenicity of this species is unknown. It constitutes a new rickettsial genotype and forms a separate cluster among the SFG rickettsiae (*3*), close to strain Cooleyi, which was isolated from *I. scapularis* in Texas (*8*). *I. ricinus* is well established in areas of northern Spain (*9*), where MSF-like cases are increasingly reported.

Our study aim was to identify the SFG rickettsial species involved in MSF-like rickettsioses in 2 patients in northern Spain. We report an association between *R. monacensis* and these rickettsioses.

## The Study

Patient 1 was an 84-year-old man from La Rioja, who sought medical attention on June 19, 2003, 7 days after onset of fever (39.5ºC), general discomfort, headache, and joint pain. At the time of the physical examination, he had a nonpruritic, disseminated maculopapular rash, with no inoculation eschar, of the trunk and lower extremities, including palms and soles. Other than a slightly low platelet count (82,000/mm^3^), examination findings were within normal limits. MSF was diagnosed, and serum and defibrinated blood samples were taken before a course of oral doxycycline (100 mg/12 h for 10 d) was initiated. Three days later, fever and rash were gone without sequelae. Additional serial serum samples were taken during weeks 4, 13, and 26 after onset and reserved for serologic analysis (Table).

**Table:** Microimmunofluorescence titers obtained with different rickettsial antigens, 2 patients, northern Spain, 2003*

Patient	Week†	*Rickettsia conorii*	*R. monacensis*	*R. helvetica*	*R. akari*	*R. australis*
1	1	<1:40	<40	<40	<40	<40
	4	1,280	2,560	2,560	1,280	640
	13	1,280	1,280	1,280	1,280	1,280
	26	1,280	1,280	1,280	320	320
2	1	640	2,560	2,560	640	320
	4	320	1,280	1,280	320	160
	6	640	1,280	1,280	320	160

*A 1-fold decrease in titer is considered not significant.  †Week after symptom onset in which the samples were extracted.

Patient 2 was a 59-year-old woman from Basque Country, who sought medical attention on September 20, 2003, 4 days after onset of fever (38ºC), headache, and an erythematous rash, with no inoculation eschar, at the site of a tick bite. The patient reported a history of tick bites, most recently 1 week before symptom onset. Blood cell counts and other blood chemistry values were normal. MSF was diagnosed, and oral doxycycline (100 mg/12 h for 10 d) was prescribed. Serial serum samples were taken the day of the visit and weeks 4 and 6 after onset and were reserved for serologic analysis (Table). Defibrinated blood was also taken 2 days after treatment was initiated. The patient recovered without sequelae.

DNA was extracted with the QIAGEN Tissue kit (IZASA S.A., Barcelona, Spain), and an *ompA*-nested PCR was designed. The first set of primers (Rr190.70p and Rr190.602n) have been described (*10*). Those used for the nested amplification were designed in this study: NompA-F (5′-AGC GAT AAT GCT GAG TAG TAG-3′) and NompA-R (5′-TAT ATT TCC TAA ACC TGT ATA A-3′) nucleotide positions 150–170 and 576–555, respectively, were numbered according to Regnery et al. (*10*). Amplification conditions were as described, except annealing temperature was 40ºC for the second PCR and AmpliTaq Gold DNA Polymerase (Applied Biosystems, Branchburg, NJ, USA) was used. The specificity of the method was tested against DNA obtained from Vero cells and *Coxiella burnetii*, and fragments of the expected sizes (532 and 427 bp) were obtained from different rickettsia species (data not shown). The amplicons obtained from blood samples were run in 1% low-melt agarose gels (Pronadisa, Barcelona, Spain), and the bands of interest were excised, purified with QIAquick Gel Extraction kit (IZASA S.A), and sequenced as described (*9*).

A phylogenetically informative fragment of 446 bp of *gltA* was also sequenced from samples by nested PCR with primers designed for this study: GLTA1F (5′-GAC GGT GAT AAA GGA ATC TTG-3′) and GLTA1R (5′-CAT TTC TTT CCA TTG TGC CAT C-3′) for the first run, and GLTA2F (5′-CTA CGA ACT TAC CGC TAT TAG-3′) and GLTA2R (5′-GAC CAA AAC CCA TTA ACC TAA AC-3′) for the second; nucleotide positions 279–299, 1011–989, 566–586, and 1298–1277, respectively, were numbered according to Regnery et al. (*10*). PCR conditions included annealing temperatures of 65ºC and 50ºC for the first and second runs, respectively. The rest of the parameters were identical to those used above, and samples were subjected to 35 cycles of denaturing (20 s at 95ºC), annealing (30 s), and extension (2 min at 60ºC), with an initial denaturing cycle of 9 min at 95ºC.

Blood samples from each patient were cultured by using shell vial technique (*11*). Giménez stain and PCR, performed after 7 days of incubation, confirmed the growth of a *Rickettsia*-like organism (strain Rp-Sp1) from patient 1. The sequences of *ompA* and *gltA* of this isolate (GenBank accession nos. DQ157778 and DQ517498, respectively) were identical to those obtained from the blood samples of each patient and to that of *R. monacensis* (*6*) (GenBank accession nos. AF201329 and DQ100163). The sequences generated in this study were subjected to phylogenetic analyses as described (*9*) and belonged to the same clade as *R. monacensis* and other related strains that have been detected in *I. ricinus* (*3*,*4*,*12*) (Figure).

**Figure:**
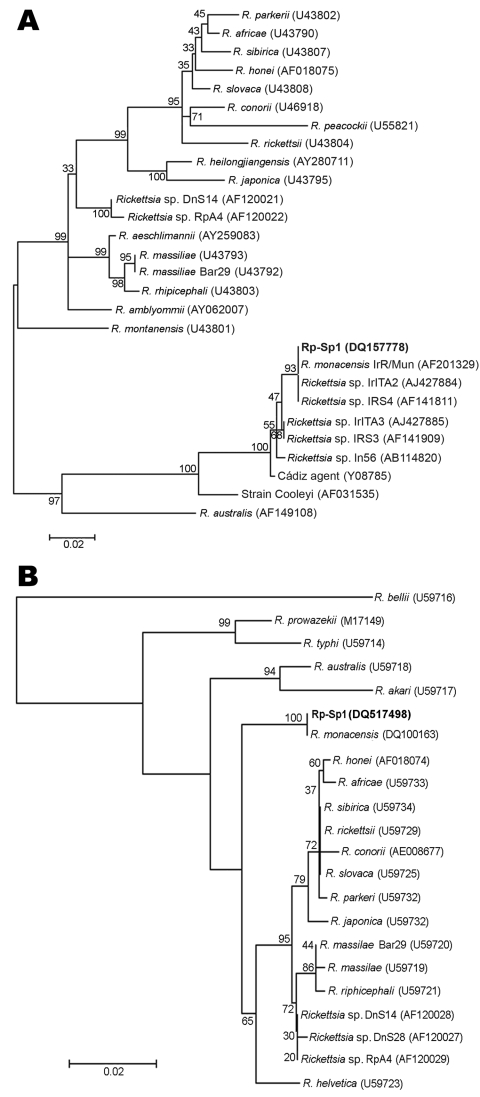
Neighbor-joining phylogenetic analysis based on *ompA* (panel A) and *gltA* (panel B). Mega 3 software (www.megasoftware.net) was used for the calculation of pairwise distances. Numbers near each node represent the bootstrap values. The isolate from patient 1 is shown in **boldface**. GenBank accession no. for each sequence is in parentheses.

In-house microimmunofluorescence assay (IFA) ([*1*] and references therein) that used *R. monacensis, R. conorii, R. helvetica, R. akari,* and *R. australis* as antigens was performed in serial serum samples from each patient (Table). The isolate Rp-Sp1 from patient 1 could not be used as antigen because of poor adaptation of this isolate to culture in Vero cell monolayers; *R. monacensis* slides for IFA were obtained from the Department of Entomology, University of Minnesota, Minneapolis, MN, USA. Seroconversion against the 5 rickettsia species was observed from patient 1’s second serum sample (day 30 after the onset). Patient 2’s first serum sample also had high titers against the 5 antigens. Although the reactivity against the 5 rickettsial antigens was similar, the titers observed were slightly higher against *R. monacensis* and *R. helvetica*, which are phylogenetically closer to each other than to the other species tested. However, because the serologic results may only loosely implicate a given rickettsia species, isolation of *R. monacensis* from patient 1 and its detection by PCR for both patients confirm it as the etiologic agent.

## Conclusions

We describe a new, to our knowledge, rickettsia species that caused human disease. *R. monacensis* was the etiologic agent of MSF-like illness in northern Spain. Strain Rp-Sp1 was obtained from 1 patient. Because the sequences of *ompA* and *gltA* were identical to this rickettsia species and also amplified from blood samples of each patient studied, we conclude that this rickettsia is responsible for the symptoms observed in these patients. Therefore, *R. monacensis* joins the list of autochthonous rickettsia species (*R. conorii* [*13*], *R. slovaca* [*14*], *R. typhi* [*15*]) confirmed as human pathogens in Spain.

We were not able to study the vectors involved; however, each patient contracted the disease in areas where *I. ricinus* is the most prevalent tick species (*9*), and strains close to *R. monacensis* have been recently detected in *I. ricinus* in Spain (*2*,*12*). Thus, *I. ricinus* may eventually be shown to be the vector. Studies of *R. monacensis* incidence in autochthonous *I. ricinus* specimens are in progress to evaluate the risk of its transmission to humans.
